# The Early Women in Dentistry

**Published:** 1894-01

**Authors:** Lucy Hobbs Taylor


### COMMUNICATIONS.

### The Early Women in Dentistry.

BY LUCY HOBBS TAYLOR, D.D.S.

Contributed at the request of Mary Gage Day, M.D., Chairman of Kansas Branch of Medical Department of Queen Isabella Association.

Somewhere back in the early part of the 50's, to be accurate, in the fall of 1859, there appeared in the western horizon a cloud "not as big as a man's hand," for it was the hand of a young girl risen in appeal to man, not for charity, but for the opportunity to enter a profession where she could earn her bread not alone by the sweat of her brow, but by the use of her brain also.

The cloud though small was portentous. It struck terror into the hearts of the community, especially the male part of it. All innovations cause commotion. This was no exception. People were amazed when they learned that a young girl had so far forgotten her womanhood as to want to study dentistry. After a lapse of almost thirty-five years it is impossible to give a just conception of the bitter opposition and the foolish objections that Lucy Hobbs had to meet and overcome.

The main objection was that her place was at home, taking care of the house. They forgot, nay worse, some of them did not care, that she had no home—and that was the main reason she wanted to learn dentistry. The search for a place to study began. She went from office to office. Some were afraid their characters would be ruined if it were known that they had a lady student, forgetting that nine-tenths of their patrons were women. One was kind enough to propose to let her come and clean his office and look on while he worked, if she would not let any one know that she was learning. This she indignantly refused. She was willing and anxious to work, but she was a self-respecting woman. Nothing daunted, she kept on until nearly every office in the city of Cincinnati, Ohio, was besieged. At last she was successful. Dr. Samuel Wardle, a large-hearted, Christian gentleman, gave her a place in his office, on the same footing as other students. To him alone belongs the honor of making it possible for women to enter the profession. He was to us what Queen Isabella was to Columbus; may his name, like hers, be revered by every woman in the profession.

No pen can portray the toil and privation of the next few months. In a little attic room the nights were spent with the needle, earning a few pennies for the morning meal; the days, incessantly toiling at a new and strange task. A previous study of medicine made the study easy, but there was much other hard work connected with an office experience. It required all her Yankee perseverance and pluck to hold on. Days, weeks and months rolled on. She was prepared for college. In March 1861, she made application to the Ohio Dental College for admission. Such boldness in a woman shocked the professors of so respectable an institution. Of course they refused.

Dr. Wardle advised her to commence practice without a diploma, as a large majority of the male practitioners of that time were not graduates. She was thwarted but not defeated, for she then fully determined never to rest until dental colleges were open to women.

She opened an office in a room in a little plain building on Fourth Street, Cincinnati, Ohio. She was of good courage even though the pittance left after rent was paid was very meagre—only twenty-five cents per week, at times, kept her from starvation.

The war commenced that April. Business was paralyzed. A penniless girl could not live where old practitioners failed. So she went to Northern Iowa. A friend helped her with money to pay her fare. She opened an office. Many called from curiosity. Little by little her practice increased. The first year she made her expenses and the one hundred dollars which paid for her dental chair. The beginning of the second year found her equipped for business.

There are passages in all lives where it would be well if all books were closed. Words never explain the heart-aches. Such a one is the lone fight of a young girl, against the whole world. Justice comes so slowly.

With the same steadfast purpose she pushed on. The second year brought reward, as the world reckons it. There was a three thousand dollar balance on the other side of the ledger. From that time on the sky was brighter. The silver lining began to show, and a confidence born of success took the place of sadness. Her reputation widened until all Iowa knew of *the woman that pulled teeth.*

The Iowa Dental Association was composed of grand, just men. Through their President, Dr. L. C. Ingersoll, they sent her an invitation to attend their convention. It was with many misgivings that the best frock was donned, the office closed and the invitation accepted; but one grasp of the hand of the president dispelled all alarm. Professional recognition after six years struggle was a balm for many old wounds. The by-laws were changed to meet the case, and the woman dentist was made a member of the association. Then the battle commenced in earnest—the woman dentist with her well-filled purse and the State of Iowa to back her, was a different person from the penniless girl with an over-weening ambition to do men's work.

She was sent with the Iowa delegates to the American Dental Association assembled at Chicago; and there met the different professors of colleges. The Iowa dentists made a formal demand for her admission to college, supported by a threat to withdraw the influence of the State from the one that refused it. The Ohio Dental College granted the request, and she entered the same fall.

Many misgivings were felt by her women friends who feared she could not stand the strain. But she went through with credit to herself and womankind. Professor J. Taft, now of the Michigan State University, then Dean of the Ohio Dental College, wrote in 1886 to the editors of the History of Woman's Suffrage concerning Miss Hobbs: "She was a woman of great energy and perseverance, studious in her habits, modest and unassuming; she had the respect and kind regard of every member of the class and faculty. As an operator she was not surpassed by her associates. Her opinion was asked and her assistance sought in difficult cases, almost daily by her fellow students. And though the class of which she was a member was one of the largest ever in attendance, it excelled all previous ones in good order and decorum—a condition largely due to the presence of a lady. In the final examination she was second to none."

Dr. George Watt, Professor of Chemistry in her Alma Mater, writes of Dr. Lucy Hobbs Taylor, "She is a credit to the profession of her choice and an honor to her Alma Mater. A better combination of modesty, perseverance and pluck is seldom if ever seen."

So the first battle was fought and won. But mark the contrast between the close, conservative East and the broad, generous West. The Eastern professors wanted to change their by-laws so that the women could not attend their associations. The Western professors attacked them through the dental journals and they had to yield the point. Then and there woman's recognition in the dental profession was made complete.

For eight years Dr. Lucy Hobbs Taylor was the only woman dentist in the world. But the dental journals relating her experience crossed the ocean. By these the desire of a German woman in Berlin to study dentistry strengthened into purpose, and Henriette Hirschfeld crossed the waters for the opportunities her native land denied her. She came to Philadelphia supposing that all colleges were open to women. She was disappointed.

However, *she* had wealthy friends who were also influential, and admission was reluctantly granted by the Pennsylvania College of Dental Surgery, from which she graduated in 1869. She returned to Germany and has been a successful practitioner, as well as a noble woman.

Shameful as it is to relate, the truth is that the students, at first, were very ungentlemanly in their treatment of Miss Hirschfeld, but they were finally won by her worth, and ladylike deportment. As late as 1872 and 1873 women were mistreated at this college. But that time has passed and most dental colleges in the United States admit women.

The struggles of these first women opened the way to a lucrative profession now practiced by several hundred women in the United States, as well as by a large number in foreign countries.

